# Isolated Zinc Deficiency Causing Severe Microcytosis and Sideroblastic Anemia

**DOI:** 10.4274/Tjh.2012.0145

**Published:** 2014-09-05

**Authors:** Gupta Shweta, Jain Prantesh, Sukhal Shashvat

**Affiliations:** 1 John H Stroger Jr Hospital of Cook County, Department of Hematology-Oncology, Chicago, United States

**Keywords:** Microcytosis, Sideroblastic anemia, Zinc deficiency

## CLINICAL PICTURES IN HEMATOLOGY

A 59-year-old Middle Eastern man with no significant past medical history was referred to the hematology clinic of our hospital for incidentally detected anemia on routine blood examination. When seen, he complained of chronic mild fatigue for 1 year. The rest of the review of systems was unremarkable. He did not take any medications and did not smoke or drink alcohol. His diet was significant for his daily intake of liver. Physical exam showed normal vital signs and was otherwise unremarkable except for pallor.

**Figure 1:**
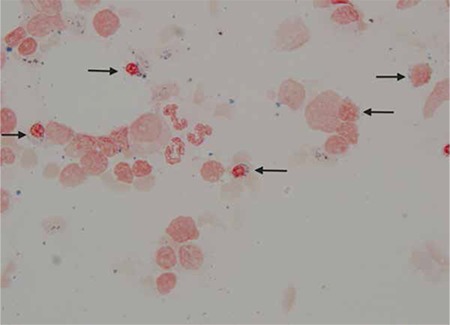
Bone marrow iron stain showing increased iron with ringed sideroblasts.

**Figure 2:**
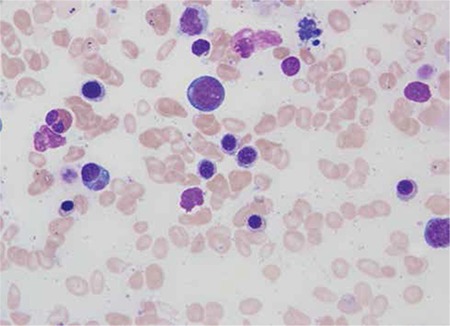
Bone marrow aspiration showing erythroid hyperplasia.

A complete blood count revealed hemoglobin of 73 g/L, total RBC count of 4.41x1012/L, MCV of 54.2 fL (low), MCH of 16.5 pg (low), MCHC of 304 g/L, RDW of 29.9%, and platelet count of 262x109/L. The reticulocyte count was 2.3% with a low reticulocyte production index, suggesting a hypoproliferative anemia. Serum ferritin level was 1336 ng/mL (normal: 24-336 ng/mL), serum vitamin B12 level was 302 pg/mL (normal: 180-914 pg/mL), and folic acid level was 4.5 ng/mL (normal: 3.5-16.1 ng/mL). A hemoglobin electrophoresis test was normal and the sickle test was negative. Erythrocyte sedimentation rate was 2 mm/h. A bone marrow aspiration/biopsy was performed, which showed a hypercellular marrow with trilineage hematopoiesis with maturation. There was microcytic anemia with erythroid hyperplasia, increased stainable iron, and 60% ringed sideroblasts, consistent with sideroblastic anemia (Figures 1, 2). There was adequate granulopoiesis and megakaryopoiesis. The flow cytometry of the bone marrow sample was negative. Fluorescent in situ hybridization was negative for chromosomal abnormalities and cytogenetic analysis showed a normal male karyotype. Based on the above findings, a working diagnosis of myelodysplastic syndrome (MDS; refractory anemia with ringed sideroblasts) was made. It was decided to observe his counts on a regular basis. Informed consent was obtained.

In the meantime, the patient started taking over-the-counter micronutrient and mineral supplements that contained copper and zinc amongst others. At the 6-month follow-up when a complete blood count was repeated, it showed a much-improved hemoglobin level of 127 g/L with MCV of 80.4 fL and normal MCH of 26.3 pg, raising the possibility of a reversible process causing sideroblastic anemia. Since the counts had corrected to near normal, he was observed. On the subsequent visit 6 months later, the patient was seen in the clinic, and he had not been taking his micronutrient and minerals pills for 3 weeks. His hemoglobin fell to 107 g/L and MCV to 66.3 fL. At that time, a test of copper and zinc levels was done, which revealed low zinc levels of 129.99 µmol/L (normal: 154.75-495.2 µmol/L) and a normal copper level of 1.4 µmol/L (normal: 1.3-2.89 µmol/L). The mineral pill was restarted, and his hemoglobin improved to 117 g/L and MCV to 73.8 fL after 2 months. He continues to be asymptomatic with stable blood counts.

